# Effect of Allelic Variations in *Wx* and *SSIIa* on Rice Cooking and Eating Quality

**DOI:** 10.3390/ijms27062588

**Published:** 2026-03-11

**Authors:** Yi Peng, Yuqianqian Li, Lin Zhang, Jing Yu, Jianming Pan, Bowen Deng, Jinsong Bao

**Affiliations:** 1 Institute of Nuclear Agricultural Sciences, Key Laboratory of Nuclear Agricultural Sciences of Ministry of Agriculture and Rural Affairs, College of Agriculture and Biotechnology, Zhejiang University, Hangzhou 310058, China; 22216121@zju.edu.cn (Y.P.); liyuqianqian@zju.edu.cn (Y.L.); dalin@zju.edu.cn (L.Z.); yuj12416018@zju.edu.cn (J.Y.); jianmingpan@zju.edu.cn (J.P.); bwdeng@zju.edu.cn (B.D.); 2 Hainan Institute, Zhejiang University, Yazhou Bay Science and Technology City, Yazhou District, Sanya 572025, China

**Keywords:** rice, starch, *Wx*, *SSIIa*, rice grain quality

## Abstract

The cooking and eating quality (CEQ) of rice is primarily regulated by the *Wx* and *SSIIa* genes. Multiple allelic variations in these genes exist in rice, but the effect of allelic combination of *Wx* and *SSIIa* on rice CEQ was less understood. In this study, the *Wx* and *SSIIa* genes of 164 rice accessions were sequenced, and the effects of nucleotide variation, both individually and in combination, on physicochemical properties such as apparent amylose content (AAC), gelatinization temperature (GT), pasting viscosities and gel texture were analyzed. Six *Wx* alleles were identified, with the highest AAC found in the *Wx^lv^* allele and the lowest in the *wx* allele. No significant difference in AAC for the same genotype harvested from two locations, Hangzhou and Sanya, was observed. Three *SSIIa* alleles were identified, i.e., G/GC, G/TT, and A/GC. The genotype with the G/GC exhibited significantly higher GT than those with G/TT and A/GC genotypes. However, the GT of the same genotype was higher in Hangzhou than in Sanya, suggesting an environmental effect. Under the same *Wx^lv^* allele background, the gel hardness (HD) of G/TT allele of *SSIIa* was the highest among all combinations, and significantly higher than that of G/GC. Under the same *wx* allele background, the peak viscosity (PV), hot paste viscosity (HPV), and cold paste viscosity (CPV) of G/GC were significantly higher than that of G/TT and A/GC. Under the same G/GC allele of *SSIIa* background, *Wx^lv^* had a slightly lower peak temperature (Tp) and a slightly higher enthalpy of gelatinization (ΔHg) than other allele combinations. Under the same G/TT allele of *SSIIa* background, *Wx^b^* had a significantly lower onset temperature (To), Tp and conclusion temperature (Tc) than other combinations. This study indicated that variation in the *Wx* gene primarily affects AAC, viscosity and gel texture, with its interaction with SSIIa influencing GT, while variation in the *SSIIa* gene primarily affects GT, with its interaction with *Wx* influencing pasting viscosity.

## 1. Introduction

Rice (*Oryza sativa* L.) is a staple food for more than half of the global population, and its quality, particularly cooking and eating quality (CEQ), directly influences consumer preferences and market value [1]. Starch, accounting for approximately 90% of the dry weight of rice endosperm, is the primary determinant of CEQ, with its structure and composition regulated by a series of key genes [1,2]. Among these, the *Waxy* (*Wx*) gene and starch synthase IIa (*SSIIa*) gene are the two most critical regulators, controlling amylose content (AAC) and gelatinization temperature (GT), the core indices of rice starch quality, respectively [2,3].

The *Wx* gene, located on rice chromosome 6, encodes granule-bound starch synthase I (GBSSI), which is the enzyme solely responsible for amylose biosynthesis in rice endosperm [4]. Natural allelic variations in the *Wx* gene are the main drivers of AAC diversity in rice germplasm. To date, multiple *Wx* alleles have been identified, including *Wx^a^*, *Wx^b^*, *Wx^lv^*, *Wx^la/mw^*, and *wx* [2,3]. The *Wx^a^* allele, predominantly present in indica rice, has a single nucleotide polymorphism (SNP) of the G nucleotide at the 5′-splicing site of the first intron, ensuring efficient intron splicing and high GBSSI expression, thus leading to high AAC (25–30%) [4]. In contrast, the *Wx^b^* allele, common in japonica rice, has a T nucleotide at the same splicing site, resulting in reduced splicing efficiency, lower GBSSI levels, and moderate AAC (15–20%) [5]. The *Wx^lv^* allele, characterized by a C-to-T mutation in the 10th exon (Ex10-115) that results in an amino acid substitution from proline to serine (Pro/Ser), exhibits high AAC similar to *Wx^a^* but produces starch with lower viscosity, contributing to unique texture properties [3]. The *Wx^la/mw^* allele, a recently identified variant, is associated with low AAC (11–14%) and transparent endosperm, making it a valuable resource for breeding high-quality soft rice [6,7]. The *wx* allele, found in glutinous rice, contains a 23 bp insertion in the second exon, leading to non-functional GBSSI and AAC below 2% [5]. These allelic variations not only affect AAC but also exert pleiotropic effects on starch viscosity, thermal properties, and textural characteristics [8,9]. Although two rice cultivars exhibit similar AAC, they still show significant differences in CEQ, which may be attributed to variations in gel consistency [10]. Studies have shown that there is a significant negative correlation between AAC and gel consistency in rice [11,12].

The *SSIIa* gene (also known as *ALK*), located on chromosome 6, encodes a soluble starch synthase IIa that participates in the elongation of medium-length chains of amylopectin, thereby regulating GT, a key trait affecting rice cooking energy and time requirements [13,14]. Three major allelic variations (SNPs) of *SSIIa* have been documented: G/GC, G/TT, and A/GC. The G/GC allele is associated with high GT (75–79 °C), while G/TT and A/GC are linked to low GT (55–69 °C) [15,16]. The functional difference between these alleles stems from non-synonymous mutations in the coding region. For instance, a G-to-A mutation at position 737 of the 8th exon (Ex8-737) that results in an amino acid substitution from valine to methionine (Val/Met) reduces SSIIa enzyme activity, leading to a decrease in medium-length amylopectin chains and thus lower GT [14]. Additionally, a 9 bp deletion in the promoter region (741 bp upstream) of the G/GC allele (designated as −9/G/GC) has been reported to further modify starch properties, such as increasing AAC, setback (SB), and hardness, while decreasing breakdown (BD) and cohesiveness (COH) compared to the wild-type G/GC allele [17]. Beyond GT, *SSIIa* also influences other starch traits, including AAC, peak viscosity (PV), and hot paste viscosity (HPV), indicating its coordinated role with the *Wx* gene in shaping starch quality [18].

The interaction between *Wx* and *SSIIa* alleles adds another layer of complexity to rice starch quality regulation. For example, under the same *SSIIa* background, *Wx^a^* genotypes typically exhibit higher AAC and SB but lower BD than *Wx^b^* genotypes [16]. Conversely, under the same *Wx* background, G/GC-type *SSIIa* results in higher GT and HPV compared to G/TT or A/GC types [18]. Moreover, environmental factors can induce genotype by environment (G × E) interactions, affecting the expression of *Wx* and *SSIIa* and then affecting starch properties and CEQ. For instance, by comparing the quantitative trait loci (QTLs) mapped at the two experimental sites of (SY) and Hangzhou (HZ), the existence of genotype-by-environment interaction effects was confirmed, and the results indicated that the RVA profile characteristics of rice were significantly affected by environmental factors [11].

Despite extensive research on individual *Wx* or *SSIIa* alleles, the combined effects of their diverse allelic combinations and G × E interactions on starch physicochemical properties remain not fully elucidated, especially in a large and diverse rice germplasm panel. In this study, 164 rice accessions were planted in Hangzhou (HZ), Zhejiang province and Sanya (SY), Hainan province to (1) identify allelic variations in *Wx* and *SSIIa*; (2) analyze the effects of these alleles and their combinations on starch traits (AAC, viscosity, GT, texture, thermal properties); and (3) evaluate G × E interactions on these traits. The results will provide insights for marker-assisted selection (MAS) in high-quality rice breeding.

## 2. Results

### 2.1. Allelic Variations in Wx and SSIIa Genes

#### 2.1.1. Polymorphism of *Wx* Gene Sequence

**Figure 1:**
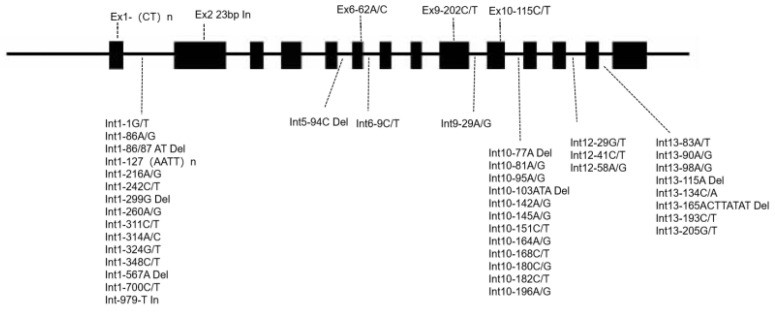
Schematic representation of the structure and polymorphic variation sites of the rice *Wx* gene. Ex: exon, Int: intron. The number following Ex and Int indicate the order of exon or intron, and the second number indicates the position of the single nucleotide polymorphism (SNP) in the respective exon or intron.

A total length of 4933 bp spanning the *Wx* gene (LOC_Os06g04200) were sequenced. Within the introns, a total of 31 SNPs and nine insertion/deletions (InDels) were identified. In the exons, there were three SNPs, which were located in the sixth, ninth, and 10th exons respectively. Additionally, a 23 bp sequence insertion was detected in the second exon (Table S1; Figure 1). The A/C SNP at the position 62 bp of the sixth exon (Ex6-62A/C) encodes amino acid changing from tyrosine to serine. The C/T SNP at the position 202 bp of the ninth exon (Ex9-202C/T) encodes a synonymous amino acid proline. However, the C/T SNP at the position 115 bp of the 10th exon (Ex10-C/T) encodes a non-synonymous amino acid (proline/serine). Additionally, (CT)n polymorphism was detected in the first exon, and a total of 10 types of (CT)n microsatellite sequences (with *n* = 8, 10, 11, 12, 16, 17, 18, 19, 20, and 21) were identified across all materials (Figure 1). An (AATT)n (*n* = 5 or 6) microsatellite was identified nearby the (CT)n (Figure 1; Table S1).

**Figure 2:**
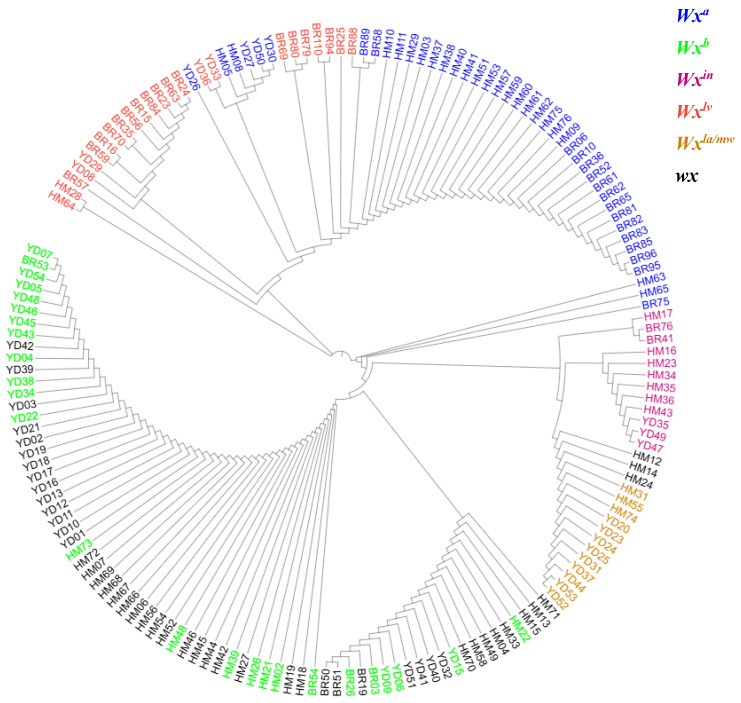
Circular phylogenetic tree of the rice *Wx* gene based on the full-length sequences from 164 rice accessions.

Based on combinations of different polymorphic sites in the *Wx* gene, nine distinct genotypes (*wx*, *Wx^a^*, *Wx^in^*, *Wx^b^*, *Wx^la/mw^*, *Wx^mp^*, *Wx^mq^*, *Wx^op/hp^*, and *Wx^lv^*) can be classified [2]. Using the full-length sequence of the *Wx* gene, we constructed a phylogenetic tree via the neighbor-joining (NJ) method implemented in MEGA7.0 (Figure 2). As shown in Figure 2, six *Wx* alleles were identified: *Wx^a^* (40 accessions), *Wx^in^* (12), *Wx^b^* (34), *Wx^lv^* (26), *Wx^la/mw^* (12), and *wx* (40). Nucleotide diversity (π) of *Wx* was 0.00286, Tajima’s D = 3.31859 (*p* < 0.01), indicating balancing selection. Non-synonymous/synonymous substitution ratio (Ka/Ks) was 2.5392, suggesting positive selection on coding regions.

#### 2.1.2. Polymorphism of *SSIIa* Gene Sequence

**Figure 3:**
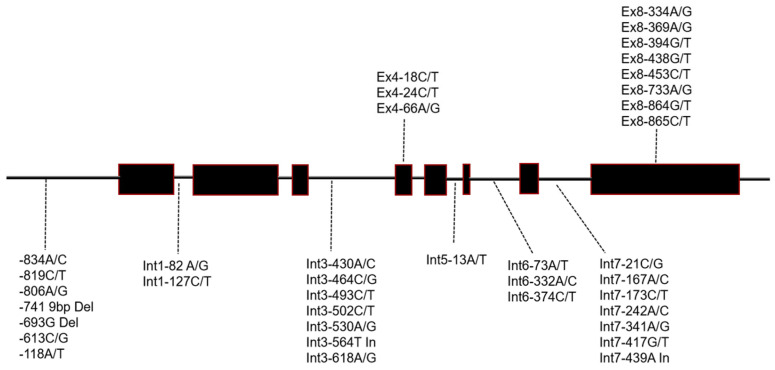
Schematic representation of the structure and polymorphic variation sites of the rice *SSIIa* gene. Ex: Exon, Int: intron, the number following Ex and Int indicate the order of exon or intron, the second number indicates the position of the single nucleotide polymorphism (SNP) in the respective exon or intron.

A total length of 5256 bp around the *SSIIa* gene (LOC_Os06g12450) were sequenced. In the promoter region, a total of five point mutations and two deletion mutations were identified, including a 9 bp deletion at the −741 bp position upstream of the promoter (Table S1; Figure 3). Within the introns, 18 point mutations and two insertion mutations were detected. In the exons, there were 11 SNPs in total, with three located in the fourth exon and eight in the 8th exon. The C/T SNP at the position 18 bp of the fourth exon (Ex4-18C/T) encodes synonymous amino acid tyrosine. The C/T SNP at the position 24 bp of the fourth exon (Ex4-24C/T) encodes synonymous amino acid aspartic acid. The Ex4-66A/G is also a synonymous polymorphism encoding amino acid lysine, another mutation (Figure 3).

The Ex8-334A/G SNP at the position 334 bp of the eighth exon led to the change in the encoded amino acid glycine to serine. The Ex8-369A/G SNP at the position 369 bp of the eighth exon encodes synonymous amino acid glutamic acid. The Ex8-394G/T SNP resulted in the change in the encoded valine with leucine. The Ex8-438G/T, Ex8-453C/T and Ex8-864G/T SNPs did not change amino acids and all encode glycine. The Ex8-733A/G SNP at the position 733 bp of the eighth exon caused the change in encoded methionine to valine. The Ex8-865C/T SNP led to the transformation of the encoded leucine to phenylalanine (Figure 3).

**Figure 4:**
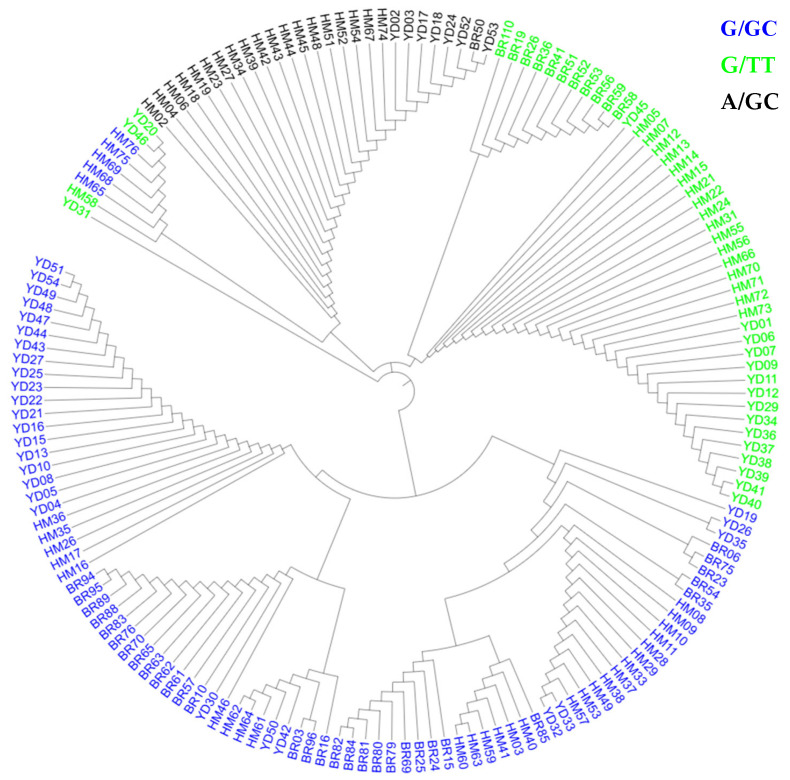
Circular phylogenetic tree of the rice *SSIIa* gene based on the full-length sequences from 164 rice accessions.

Based on the allelic combinations at the Ex8-4198 locus (polymorphic for G/A) and Ex8-4329/4330 locus (polymorphic for GC/TT), the *SSIIa* gene can be categorized into three distinct genotypes: G/GC, G/TT, A/GC [2]. To illustrate the genetic relationships among the *SSIIa* genotypes, a circular phylogenetic tree was constructed based on the full-length gene sequence, using the neighbor-joining (NJ) method in MEGA7.0. The tree clusters the materials into three distinct clades corresponding to the three *SSIIa* genotypes:G/GC (91 accessions), G/TT (47), and A/GC (26) (Figure 4). According to the report by Zheng et al., a 9 bp insertion/deletion(InDel) was identified at the position 741 bp of the *SSIIa* promoter. Such a 9 bp InDel occurs only in the G/GC genotype [17]. Therefore, to investigate the function of this mutation, we designated the genotype of materials carrying the 9 bp InDel within the G/GC haplotype as −9/G/GC.In this study, a subset of G/GC (40 accessions) had a 9 bp promoter deletion. *SSIIa* π was 0.00234, Tajima’s D = 3.04887 (*p* < 0.01), and Ka/Ks = 0.1923, indicating purifying selection.

### 2.2. Genetic Diversity of the Starch Physicochemical Properties

Although 121 accessions were grown in Hangzhou, only 76 accessions were harvested with enough seeds for analysis, while others failed to flower due to an unsuitable photoperiod. All 164 accessions grown in Sanya were harvested with enough seeds for quality analysis. Table 1 presents the statistical parameters of phenotypic traits of the tested materials under two environments (HZ and SY), including range, mean, coefficient of variation (CV), skewness, and kurtosis. The large ranges and CV indicated wide variation among different genotypes across the two environments. In the HZ environment, AAC ranged from 1.0 to 29.7% with a mean of 14.3% and a high CV of 75.5%. In the SY environment, the AAC had a slightly wider range (0.3–31.5%) and higher mean (16.1%) than in HZ, with a lower CV (63.8%). For viscosity-related traits, the peak viscosity (PV) had a range of 41.2–373.6 RVU and a mean of 226.3 RVU, while the hot paste viscosity (HPV) and cool paste viscosity (CPV) averaged 129.0 and 207.0 RVU with CVs of 46.7% and 52.9% in HZ. In SY, the PV averaged 220.4 RVU with a range of 64.5–381.9 RVU and a CV of 29.4%, and the HPV had a higher mean (141.6 RVU) and lower CV (33.4%) compared to HZ. The BD showed a higher skewness (1.29) and CV (53.7%) in SY than in HZ. The setback (SB) showed the highest CV among all traits in both environments. For thermal properties, the onset temperature (To), peak temperature (Tp), and conclusion temperature (Tc) had narrow CVs (3.9–6.4%) in both environments. In HZ, the gel textural properties of eight accessions were not detected. The hardness (HD) and gumminess (GUM) exhibited relatively high coefficients of variation (CVs) in HZ (103.6% and 99.2%, respectively), while those in SY showed lower CVs (85.0% and 85.3%) compared to HZ.

### 2.3. Correlation Analysis

**Figure 5:**
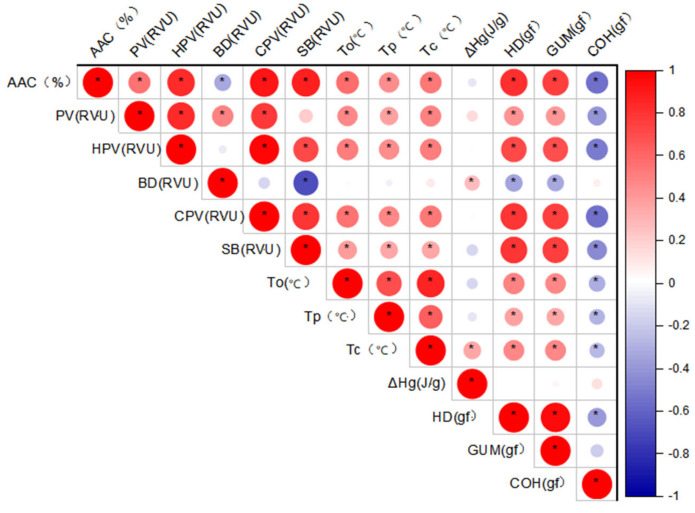
Pearson correlation coefficient heatmap of rice starch physicochemical properties under Hangzhou planting conditions. * indicates significance at *p* < 0.05.

Correlation analysis was performed on 13 traits related to the physicochemical properties of rice starch planted in Hangzhou in 2023. Except for ΔHg, AAC showed significant correlations with the other traits. Specifically, AAC was positively correlated with PV, HPV, CPV, SB, To, Tp, Tc, HD, and GUM, while it was negatively correlated with BD and COH (Figure 5).

**Figure 6:**
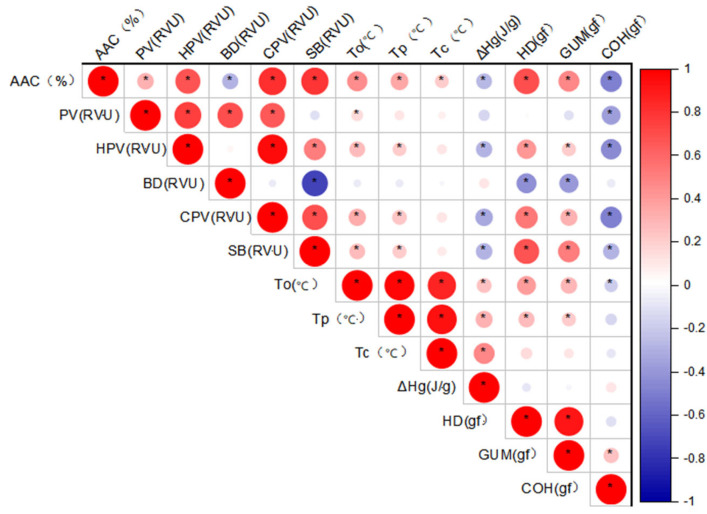
Pearson correlation coefficient heatmap of rice starch physicochemical properties under Sanya planting conditions. * indicates significance at *p* < 0.05.

Compared with the rice planted in Hangzhou in 2023, the correlation coefficients of the 13 related traits differed in the rice planted in Sanya in 2024. Specifically, AAC showed a significant negative correlation with ΔHg in Sanya. In Hangzhou, To, Tp, and Tc had no correlation with BD, but exhibited significant positive correlations with other pasting properties. In contrast, in Sanya, Tc showed no significant correlations with any of the pasting properties (Figure 6).

### 2.4. Genotype × Environment (G × E) Effects

Analysis of variance (ANOVA) was conducted on the physicochemical properties of starch of 76 accessions harvested in both environments to evaluate the effects of genotype (G), environment (E), and genotype-by-environment interaction (G × E) (Table 2; Table S2). The results showed that except for the effect of environment (E) on cohesiveness (COH) which was significant at *p* < 0.05, all other traits were significantly affected by genotype (G), environment (E), and G × E interactions at *p* < 0.001 (Table 2). AAC, CPV, and HD were mainly affected by the genotype since the genotypic variance accounted for 97.4, 93.5 and 81.5% of the total variance (Table 2). Although, gelatinization temperatures (To, Tp and Tc) were not only affected by genotype, but also by environment effect and G × E, with the effect of environment being greater than G × E. Pasting viscosities were not only affected by genotype, but also by environment effect and G × E, with the G × E effect being greater than the environment effect (Table 2).

### 2.5. Effects of Wx Alleles on Starch Physicochemical Properties

#### 2.5.1. Apparent Amylose Content (AAC)

AAC differed significantly among *Wx* alleles (*p* < 0.001) but was stable across environments (Table 3). In both HZ and SY: *Wx^lv^* and *Wx^a^* had the highest AAC (~26%); *Wx^in^* (20–22%) > *Wx^b^*/*Wx^la/mw^* (10–14%); *wx* had the lowest (<2%).

#### 2.5.2. Pasting Properties

Rice with genotype *Wx^b^* exhibited the highest peak viscosity (PV), followed by *Wx^la/mw^* and *Wx^a^*. No significant differences were observed among these three genotypes. In contrast, genotype *wx* had the lowest PV, which was significantly lower than that of the other genotypes. Regarding hot paste viscosity (HPV), genotype *Wx^a^* showed a significantly higher value than the other five genotypes, while genotype *wx* had the lowest HPV. For breakdown viscosity (BD), genotype *Wx^la/mw^* had the highest value, followed by *Wx^b^*, and *Wx^lv^* exhibited the lowest BD. In terms of cool paste viscosity (CPV), genotype *Wx^a^* had the highest value, and genotype *wx* had the lowest. Significant differences in CPV were detected among all genotypes except between *Wx^in^* and *Wx^lv^*. Additionally, genotype *Wx^a^* showed the highest setback viscosity (SB), whereas genotype *Wx^la/mw^* had the lowest SB (Table 3).

#### 2.5.3. Textural Properties

In the materials grown in Sanya, there was no significant difference in hardness (HD) between the *Wx^lv^* and *Wx^a^* genotypes, but their HD values were significantly higher than those of the other four genotypes. No significant differences in HD were observed among the remaining four genotypes. Additionally, the gumminess (GUM) of *Wx^lv^* and *Wx^a^* was significantly higher than that of the other four genotypes. Genotype *wx* exhibited the highest cohesiveness (COH), while no significant differences in COH were detected among the other five genotypes. In Hangzhou, trends were similar (Table 3).

#### 2.5.4. Thermal Properties

Among the materials grown in Sanya, the To and Tp of *Wx^a^* were the highest, while those of *wx* were the lowest. The Tc of *Wx^la/mw^* was the highest, and that of *Wx^b^* was the lowest. The enthalpy change (ΔHg) of *Wx^la/mw^* was the highest, whereas that of *Wx^in^* was the lowest. In Hangzhou, the To, Tp, and Tc of *Wx^a^* and *Wx^in^* were relatively higher, followed by *Wx^b^*, with *wx* showing the lowest values. The ΔHg of *Wx^b^* was the highest, while that of *Wx^in^* was the lowest (Table 4).

### 2.6. Effects of SSIIa Alleles on Starch Physicochemical Properties

#### 2.6.1. Thermal Properties

In both locations, GT of different *SSIIa* alleles followed: −9/G/GC ≈ G/GC (To: 72.4–76.5 °C) > G/TT (66.4–68.9 °C) ≈ A/GC (67.0–70.1 °C). GT was 2–3 °C higher in HZ than in SY for the same genotype (Table 5).

#### 2.6.2. AAC

The allele of −9/G/GC had the highest AAC (24.4–25.3%), followed by the G/GC allele (16.4–18.4%), and the allele of G/TT and A/GC allele had lowest AAC (7.1–10.8%) (Table 6).

#### 2.6.3. Pasting Properties

The *SSIIa* allele −9/G/GC and G/GC had higher PV (218.1–265.5 RVU), HPV (152.7–178.4 RVU), CPV (256.2–305.3 RVU), and SB (10.5–56.1 RVU) than the G/TT and A/GC allele. The −9/G/GC allele had the lowest BD (58.5–79.8 RVU) (Table 6).

#### 2.6.4. Textural Properties

The *SSIIa* −9/G/GC allele had the highest HD (12.1–16.1 gf) and GUM (5.5–7.2 gf), while −9/G/GC had the lowest COH (0.4 gf) (Table 6).

### 2.7. Interaction Between Wx and SSIIa Alleles

If there were more than five accessions in each allele combination of *Wx* and *SSIIa*, the starch physicochemical properties in the Sanya environment were compared. For simplicity, we used Type 1–4 to stand for −9/G/GC, G/GC, G/TT, and A/GC of the *SSIIa* gene, respectively. Under the same *Wx* background, there are no significant differences in AAC, viscosity and textural properties between Type1/*Wx^a^* and Type2/*Wx^a^* (Table 7). Similarly, no significant differences in viscosity characteristics and texture properties were observed among Type2/*Wx^b^*, Type3/*Wx^b^*, and Type4/*Wx^b^*. The HD of Type3/*Wx^lv^* was highest among all combinations, significantly higher than those of Type1/*Wx^lv^* and Type2/*Wx^lv^*. The PV, HPV and CPV of Type2/*wx* were significantly higher than those ofType3/*wx* and Type4/*wx* (Table 7).

There were no significant differences in To, Tp, Tc, or ΔHg between Type1/*Wx^a^* and Type1/*Wx^lv^* (Table 8). Among the type2 of *SSIIa*, Type2/*Wx^lv^* had a little lower Tp and a little higher ΔHg than other combinations. Among the type3 of *SSIIa*, Type3/*Wx^b^* had significantly lower To, Tp and Tc than other combinations. There was no significant difference in all the thermal properties between Type4/*Wx^b^* and Type4/*wx* (Table 8).

## 3. Discussion

### 3.1. Wx Alleles Are Key Regulators of AAC, Pasting Properties and Gel Textural Properties

This study identified six *Wx* alleles (*Wx^a^*, *Wx^in^*, *Wx^b^*, *Wx^lv^*, *Wx^la/mw^*, and *wx*) in 164 rice accessions, confirming their pivotal role in shaping amylose content (AAC) diversity, a finding consistent with previous research [2,3,5,6,7]. Rice accessions with *Wx^lv^* and *Wx^a^* alleles exhibited the highest AAC (≈26%) in Sanya (SY), while in Hangzhou (HZ), *Wx^a^* also displayed the highest AAC (Table 3); this was due to the higher activity of granule-bound starch synthase I (GBSSI) in these alleles [2,3]. This high AAC directly translated to distinct textural and pasting characteristics: these alleles showed significantly higher hardness (HD, 13.6–15.8 gf) and lower breakdown viscosity (BD, 48.9–74.6 RVU) compared to other alleles (Table 3) [8,19]. The low BD value indicates reduced starch granule disintegration during cooking, resulting in a firm texture, consistent with Zhang et al. [3]. This makes *Wx^lv^* a valuable resource for breeding functional rice varieties rich in resistant starch, which offers health benefits such as improved glycemic control [20].

The newly characterized *Wx^la/mw^* allele displayed moderate-low AAC (10.9–13.8%) and exceptional pasting properties, including the highest BD (136.9 RVU) and lowest setback viscosity (SB = −85.7 RVU) (Table 3). This combination reflects its balanced GBSSI activity, enabling starch to gelatinize easily during cooking and resist retrogradation upon cooling, key attributes of high-quality soft rice. Moreover, *Wx^la/mw^* can improve ECQ and grain transparency in high-yielding japonica cultivars, and it is a promising allele for grain quality improvement in rice breeding programs [6,7].

Rice genotype with *Wx^b^* and *wx* alleles cluster together in the phylogenic tree (Figure 1), because the *wx* allele, distinguished from *Wx^b^* by a 23 bp insertion in the second exon [2], led to non-functional GBSSI and extremely low AAC (<2%). This genetic defect resulted in unique physicochemical traits: low peak viscosity (PV = 155.7 RVU) and low hot paste viscosity (HPV = 85.6 RVU) (Table 3) [21]. These observed changes in pasting properties are consistent with the findings of Fu et al. [22], who reported that CRISPR/Cas9-mediated *wx* mutants with non-functional GBSSI showed a significant decrease in AAC and viscosity.

*Wx^in^* and *Wx^b^* alleles exhibited intermediate AAC (13.2–22.1%), with *Wx^b^* showing higher PV (269.6 RVU) and BD (118.3 RVU) than *Wx^in^* (Table 3). This difference is likely due to variations in intron splicing efficiency: a T nucleotide in the first intron reduces GBSSI expression [5], leading to a potentially better swelling properties during cooking [23]. These intermediate alleles cater to diverse consumer preferences, bridging the gap between firm high-AAC rice and sticky low-AAC varieties.

### 3.2. SSIIa Alleles Are Major Determinants of Gelatinization Temperature

Three *SSIIa* alleles (G/GC, G/TT, A/GC) were identified, with consistent effects on gelatinization temperature (GT), a critical trait influencing rice cooking time and energy consumption [13,14]. A 9 bp deletion in the promoter region was uniquely detected in rice accessions with the G/GC allele [17], so a subgroup of −9/G/GC can be further resolved (Table 5). Rice with G/GC exhibited significantly higher onset temperature (To = 72.4–76.5 °C) compared to G/TT (66.4–68.9 °C) and A/GC (67.0–70.1 °C) across both environments (Table 5), consistent with previous studies [16,17,24]. Especially, Zheng et al. indicated that the gelatinization temperature of −9/G/GC (type 1 in this study) was lower than that of G/GC (type 2) [17], we found that the difference was not significant in this study (Table 5). However, the −9 bp in the promoter region can be used in molecular breeding for rice quality improvement [17], because detection of a 9 bp deletion could be easily resolved by agrose gel electrophesis, while detection of SNPs were quite difficult in agrose gel.

### 3.3. Wx × SSIIa Interaction: Implications for Quality Breeding

The interaction between *Wx* and *SSIIa* significantly influenced starch properties. *Wx* dominates AAC, pasting properties and gel textural properties, and *SSIIa* modulates GT while fine-tuning other traits [16,18,25]. To compare the starch physicochemical properties of different alleles of one gene within the same allelic background of another gene, it is easy to find the interactions between *Wx* and *SSIIa*. For example, under the same *Wx^lv^* allele background, the HD of G/TT (type3 *SSIIa*) was the highest among all combinations, and under the same *wx* allele background, the PV, HPV, and CPV of *G/GC* (type2 *SSIIa*) was significantly higher than other alleles (Table 7). Similarly, under the same G/GC (type2) allele of *SSIIa* background, *Wx^lv^* had a slightly lower Tp and a slightly higher ΔHg than other alleles, and under the same G/TT (type3) allele of *SSIIa* background, *Wx^b^* had significantly lower To, Tp and Tc than other alleles (Table 8). All the results suggested significant interactions between *Wx* and *SSIIa*. The *Wx^b^*/*SSIIa* G/GC combination had moderate AAC (13.8%), high BD (121.9 RVU), and low SB(−51.6 RVU) (Table 7), and showed better ECQ compared to other allele combinations [26]. Rice samples with different combinations of AAC and GT vary in optimal cooking temperature and time [27,28]. In this regard, rice with different combinations of *Wx* and *SSIIa* alleles require specific optimal cooking conditions. This interaction highlights the need for coordinated selection of *Wx* and *SSIIa* alleles to achieve desired quality traits.

### 3.4. Effects of Genotype × Environment (G × E) on Rice Quality

Environmental factors affected traits like PV and HPV but had little effect on AAC, indicating that AAC is genetically stable (genotype contribution accounts for 97.4%), while pasting properties are more plastic [29]. GT was 2–3 °C higher in HZ than in SY for the same genotype, likely due to differences in temperature and light during grain filling; higher temperatures promote medium-length amylopectin synthesis, increasing GT [30]. Chun et al. [28] also reported that as the ripening temperature increased, the AAC and number of short amylopectin chains decreased, whereas intermediate amylopectin chains increased, resulting in higher gelatinization temperatures and enthalpy in the starch. The significant G × E effect on SB (10.2% variance contribution) suggests that starch retrogradation is sensitive to environmental fluctuations, which may affect the shelf life and processing applicability of cooked rice. This highlights the need to select stable genotypes across environments for consistent quality [31]. The molecular markers developed from the SNPs identified in this study may accelerate the molecular selection of desired starch qualities [2,32].

## 4. Materials and Methods

### 4.1. Plant Materials and Growth Conditions

A total of 164 rice accessions were used, including 69 accessions labeled with a prefix “HM”, 52 accessions with a prefix “YD”, and 43 accessions with a prefix “BR”. Those HM and YD accessions were grown in HZ (2023) and SY (2024), while BR accessions were only grown in SY (2024) due to flowering failure under long day period in HZ. The experiment employed a randomized complete block design with two replications. Mature grains were dried to 12% moisture, stored at room temperature for 2 months, and milled into white rice and flour passing through 100-mesh sieve using a Satake TM05C miller (Houston, TX, USA), and UDY cyclone mill (Fort Collins, CO, USA), respectively.

### 4.2. DNA Extraction and Gene Sequencing

**Figure 7:**
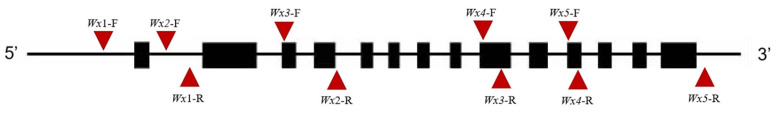
Schematic diagram of the primer design for the full-length amplification of the rice *Wx* gene.

**Figure 8:**
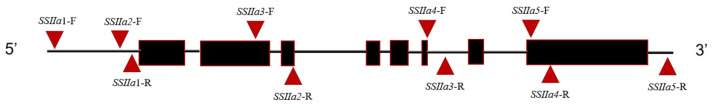
Schematic diagram of the primer design for the full-length amplification of the rice *SSIIa* gene.

Genomic DNA was extracted from 1-month-old leaves of all 164 rice accessions using the CTAB method [33]. Primers for *Wx* (LOC_Os06g04200) covering the promoter to 3′ untranslated region with a total of 4933 bp and *SSIIa* (LOC_Os06g12450) covering the promoter to 3′ untranslated region with a total of 5256 bp were designed (Figure 7 and Figure 8; Table 9). PCR amplification of the *Wx* and *SSIIa* genes was performed using all the designed specific primers (Table 9). The purified DNA fragments were sequenced by Sangon Biotech (Shanghai, China) for Sanger sequencing. The sequences were aligned using MEGA7.0 to identify allelic variations.

### 4.3. Determination of Starch Physicochemical Properties

#### 4.3.1. Apparent Amylose Content

AAC was measured by the iodine colorimetric method [34]. Rice flour (0.01 g) was mixed with 10 μL 95% ethanol and 900 μL 1 M NaOH, boiled for 10 min, diluted to 10 mL, and reacted with I_2_-KI-acetate solution. Absorbance at 620 nm was measured using a BioTek Epoch microplate spectrophotometer (Epoch, Biotek, Winooski, VT, USA), and AAC was calculated via a standard curve, which was made from five standard rice samples with known amylose content, i.e., BP595 (2.0%), BP608 (8.0%), BP025 (17.0%), SN05 (22.0%) and BP037 (28.5%).

#### 4.3.2. Pasting Properties

Pasting viscosities were determined using a Rapid Visco Analyzer (RVA, Model 4500, Perten Instrument, Hägersten, Sweden). A total of 3 g of flour were mixed with 25 g of ddH_2_O, and the RVA profile (peak viscosity [PV], hot paste viscosity [HPV], cold paste viscosity [CPV], breakdown [BD = PV − HPV], and setback [SB = CPV − PV]) were recorded using the Thermocline for Windows version 3 (TCW3) software.

#### 4.3.3. Thermal Properties

Thermal properties were analyzed using a Differential Scanning Calorimeter (DSC, Q20, TA Instruments, New Castle, DE, USA). This method was slightly modified from the protocol described by Zhao et al. [19]. First, rice flour (2 mg, with a moisture content of 12%) and water were weighed at a weight ratio of 1:3, then sealed in an aluminum crucible. Subsequently, the sealed crucible was equilibrated at 4 °C for 24 h, followed by an additional equilibration at room temperature for 1 h. After that, the crucible was placed into a differential scanning calorimeter (DSC), and held at 30 °C for 1 min. Then, the temperature of the crucible was increased from 30 °C to 110 °C at a heating rate of 10 °C/min, with an empty crucible used as the reference. Parameters including onset temperature (To), peak temperature (Tp), conclusion temperature (Tc), and gelatinization enthalpy (ΔHg) were calculated using Universal Analysis 2000 software.

#### 4.3.4. Textural Properties

Gels from the RVA test were maintained at 4 °C for 24 h, and the gel texture was measured by a texture analyzer (TA.XTC-18, Shanghai Bosin Industrial Development Co., Shanghai, China). The gels were compressed to 10 mm at 1 mm/s using a 5 mm probe. Hardness (HD), gumminess (GUM), and cohesiveness (COH) were recorded.

### 4.4. Statistical Analysis

All measurements were performed in triplicate. Data were analyzed using SAS 8.0 for analysis of variance (ANOVA) using a fixed model to quantify genotype (G), environment (E), and G × E effects. Sequence assembly of the *Wx* and *SSIIa* genes was performed using SeqMan (DNAstar) version 7.1.0. Haplotypes among different materials were identified using DnaSP v5 [35]. A phylogenetic tree of the tested materials was constructed via the neighbor-joining (NJ) method implemented in MEGA7.0 with a bootstrap value of 1000 replicates, followed by visualization and optimization with iTOL version 6 (https://itol.embl.de/, accessed on 5 February 2025) [36]. Significant differences between means were determined by Duncan’s multiple range test (*p* < 0.05).

## 5. Conclusions

This study systematically identified six alleles of the *Wx* gene and three main alleles of the *SSIIa* gene and compared their effects on the physicochemical properties among different alleles of each gene. The combined effects of *Wx* and *SSIIa* alleles on starch physicochemical properties were also analyzed. The results showed that under the same genetic background of one gene, different alleles of the other gene led to variations in starch physicochemical properties, strongly suggesting significant interactions between the two genes. Significant G × E effects were detected for all traits; however, the magnitude of these effects varied depending on the specific trait examined. The identified SNPs and the observed combined effects of different alleles provide valuable molecular markers for marker-assisted breeding of high-quality rice varieties.

## Figures and Tables

**Table 1:** Values of starch quality parameters for the rice harvested in Hangzhou (HZ) and Sanya (SY).

Environment	Trait	Range	Mean	CV (%)	Skewness	Kurtosis
HZ	AAC (%)	1.0–29.7	14.3	75.5	−0.05	−1.63
	PV (RVU)	41.2–373.6	226.3	31.9	−0.53	0.23
	HPV (RVU)	10.0–246.5	129.0	46.7	−0.12	−0.96
	BD (RVU)	29.3–236.9	97.3	41.2	0.76	1.23
	CPV (RVU)	15.6–394.2	207.0	52.9	−0.05	−1.65
	SB (RVU)	−188.0–107.2	−19.3	370.4	0.04	−1.11
	To (°C)	60.4–80.0	72.7	6.4	−0.28	−0.54
	Tp (°C)	67.2–83.7	77.6	5.2	−0.41	−0.32
	Tc (°C)	72.3–89.7	83.0	4.5	−0.23	−0.34
	ΔHg (J/g)	1.8–9.8	5.4	35.9	−0.11	−1.11
	HD (gf) ^a^	0.8–33.1	8.1	103.6	1.16	0.16
	GUM (gf) ^a^	0.5–14.6	4.1	99.2	1.23	0.18
	COH ^a^	0.2–1.0	0.6	28.9	−0.02	1.21
SY	AAC (%)	0.3–31.5	16.1	63.8	−0.24	−1.42
	PV (RVU)	64.5–381.9	220.4	29.4	0.01	−0.48
	HPV (RVU)	18.3–228.4	141.6	33.4	−0.20	−0.88
	BD (RVU)	20.7–227.5	78.8	53.7	1.29	1.32
	CPV (RVU)	26.8–401.2	225.8	39.8	−0.24	−1.10
	SB (RVU)	−177.0–126.0	5.4	1275.0	−0.28	−0.84
	To (°C)	60.5–77.8	70.0	6.2	−0.04	−1.26
	Tp (°C)	67.6–81.9	75.1	4.5	0.02	−0.94
	Tc (°C)	72.2–86.7	80.0	3.9	0.06	−0.59
	ΔHg (J/g)	1.3–10.2	6.5	23.8	−0.4	−0.01
	HD (gf)	1.2–31.7	7.8	85.0	1.20	0.61
	GUM (gf)	0.1–14.9	4.0	85.3	1.05	0.32
	COH	0.1–1.2	0.5	44.1	0.59	0.59

^a^ In HZ, only 68 accessions had available gel texture data. CV: coefficient of variation.

**Table 2:** Mean variance of genotype (G), environment (E) and G × E for starch properties from analysis of variance.

SOV	DF	AAC	PV	HPV	BD	CPV	SB	HD	GUM	COH	To	Tp	Tc	ΔHg
Genotype (G)	75	461.8 ***	13,113 ***	6115 ***	4067 ***	41,614 ***	17,371 ***	180.1 ***	39.8 ***	0.11 ***	69.9 ***	43.8 ***	35.3 ***	6.2 ***
Environment (E)	1	9.8 ***	3639 ***	8882 ***	23,891 ***	16,954 ***	36,302 ***	146.2 ***	39.7 ***	0.06 *	832.7 ***	748.2 ***	1061 ***	22.1 ***
G × E	75 (67) ^a^	12.0 ***	4452 ***	1268 ***	1633 ***	2690 ***	1912 ***	43.4 ***	22.7 ***	0.07 ***	8.4 ***	6.1 ***	8.2 ***	5.0 ***
Error	152 (144) ^a^	0.1	120	34	70	65	41	0.5	1.0	0.01	0.1	0.1	0.3	0.2

^a^ The numbers in parentheses indicate the DF for the gel texture parameters (HD, GUM, and COH), for which data from eight accessions were not detected in HZ. SOV: source of variance; DF: degrees of freedom. * and *** indicate significance at *p* < 0.05 and 0.001, respectively.

**Table 3:** AAC, pasting and textural properties of rice with different *Wx* alleles harvested in Hangzhou (HZ) and Sanya (SY).

Environments	Alleles	Number	AAC (%)	PV (RVU)	HPV (RVU)	BD (RVU)	CPV (RVU)	SB (RVU)	HD (gf)	GUM (gf)	COH
HZ	*Wx^a^*	24	25.7 a	250.7 a	176.0 a	74.6 c	305.6 a	55.0 a	15.8 a	7.3 a	0.5 b
	*Wx^in^*	6	18.6 b	253.5 a	158.0 ab	95.5 b	257.9 ab	4.4 b	5.7 b	3.0 b	0.5 b
	*Wx^b^*	12	13.8 c	280.0 a	138.1 b	149.9 a	210.2 c	−69.8 c	3.2 b	1.9 b	0.6 b
	*wx*	27	1.5 d	166.5 b	68.2 c	98.3 b	89.1 c	−77.4 c	1.8 b	1.2 b	0.7 a
SY	*Wx^a^*	40	26.1 a	248.1 ab	189.2 a	59.0 bc	323.7 a	75.6 a	13.6 a	6.1 a	0.42 b
	*Wx^in^*	12	22.1 b	223.1 b	154.3 b	68.8 bc	265.5 b	42.4 b	5.6 b	2.8 b	0.46 b
	*Wx^b^*	34	13.2 c	269.6 a	151.3 b	118.3 a	226.8 c	−42.9 c	3.5 b	1.9 b	0.49 b
	*Wx^lv^*	26	26.4 a	189.1 c	140.9 bc	48.9 c	255.1 b	65.2 a	14.5 a	6.5 a	0.44 b
	*Wx^la/mw^*	12	10.9 c	268.1 a	131.2 c	136.9 a	182.4 d	−85.7 d	3.0 b	1.4 b	0.46 b
	*wx*	40	1.5 d	155.7 d	85.6 d	70.1 b	109.3 e	−46.4 c	3.4 b	3.0 b	0.78 a

Different letters in the same column indicated significant difference at *p* < 0.05.

**Table 4:** Thermal properties of rice with different *Wx genes* harvested in Hangzhou (HZ) and Sanya (SY).

Environments	Alleles	Number	To (°C)	Tp (°C)	Tc (°C)	ΔHg (J/g)
HZ	*Wx^a^*	24	75.8 a	80.4 a	85.1 a	5.1 ab
	*Wx^in^*	6	76.0 a	80.5 a	85.0 a	4.2 b
	*Wx^b^*	12	73.7 ab	78.5 ab	84.0 a	6.5 a
	*wx*	27	69.7 b	73.7 b	80.7 b	5.5 ab
SY	*Wx^a^*	40	72.2 a	76.4 a	80.6 ab	5.9 cd
	*Wx^in^*	12	69.3 bc	75.2 abc	80.3 ab	5.6 d
	*Wx^b^*	34	68.9 bc	74.2 bc	79.4 b	6.3 bcd
	*Wx^lv^*	26	72.0 a	76.3 ab	80.8 ab	6.6 abc
	*Wx^la/mw^*	12	70.9 ab	76.1 ab	81.8 a	7.5 a
	*wx*	40	67.2 c	73.4 c	80.0 b	7.1 ab

Different letters in the same column indicated significant difference at *p* < 0.05.

**Table 5:** Thermal properties of rice with different *SSIIa genes* harvested in Hangzhou (HZ) and Sanya (SY).

Environments	Alleles	Number	To (°C)	Tp (°C)	Tc (°C)	ΔHg (J/g)
HZ	−9/G/GC	21	76.0 a	80.4 a	85.2 a	4.9 b
	G/GC	16	76.5 a	81.1 a	86.4 a	6.2 a
	G/TT	23	68.9 b	72.9 b	80.1 b	5.5 a
	A/GC	16	70.1 c	75.3 c	80.8 bc	5.2 ab
SY	−9/G/GC	40	73.2 a	77.3 a	81.5 a	6.2 b
	G/GC	51	72.4 a	77.0 a	82.1 a	6.9 a
	G/TT	47	66.4 b	72.4 b	77.8 b	6.4 ab
	A/GC	26	67.0 b	72.7 b	77.9 b	6.1 b

Different letters in the same column indicated significant difference at *p* < 0.05.

**Table 6:** AAC, pasting properties and textural properties of rice with different *SSIIa* alleles harvested in Hangzhou (HZ) and Sanya (SY).

Environments	Alleles	Number	AAC (%)	PV (RVU)	HPV (RVU)	BD (RVU)	CPV (RVU)	SB (RVU)	HD (gf)	GUM (gf)	COH (gf)
HZ	−9/G/GC	21	25.3 a	258.2 a	178.4 a	79.8 b	305.3 a	47.1 a	16.1 a	7.2 a	0.4 b
	G/GC	16	16.4 b	265.5 a	152.7 a	112.8 a	243.8 b	−21.7 b	9.1 b	4.9 b	0.6 a
	G/TT	23	8.0 c	195.6 b	90.3 b	105.4 ab	134.6 c	−61.1 c	2.5 c	1.7 c	0.7 a
	A/GC	16	7.1 c	189.1 b	96.1 b	93.0 ab	145.2 c	−43.9 bc	3.8 c	2.2 bc	0.6 a
SY	−9/G/GC	40	24.4 a	218.1 ab	159.6 a	58.5 b	274.2 a	56.1 a	12.1 a	5.5 a	0.4 c
	G/GC	51	18.4 b	245.7 a	158.6 a	87.0 a	256.2 a	10.5 b	8.3 b	4.0 b	0.5 bc
	G/TT	47	10.8 c	206.5 b	122.4b	84.1 a	183.8 b	−22.7 c	5.5 c	3.0 b	0.6 ab
	A/GC	26	8.4 c	199.8 b	115.4b	84.4 a	168.1 b	−31.7 c	4.5 c	3.2 b	0.6 a

Different letters in the same column indicated significant difference at *p* < 0.05.

**Table 7:** AAC, pasting properties and textural properties of rice with different *SSIIa* and *Wx* allele combinations.

Alleles	Number	AAC (%)	PV (RVU)	HPV (RVU)	BD (RVU)	CPV (RVU)	SB (RVU)	HD (gf)	GUM (gf)	COH (gf)
Type1/*Wx^a^*	21	26.8 a	247.2 a	183.5 a	63.7 bc	319.4 a	72.2 a	13.9 ab	6.3 ab	0.4 b
Type2/*Wx^a^*	14	25.3 a	253.0 a	199.1 a	53.9 c	332.7 a	79.7 a	13.9 ab	6.1 ab	0.4 b
Type2/*Wx^in^*	7	22.7 a	237.8 ab	154.4 abc	83.4 b	270.0 ab	32.0 b	5.3 c	2.7 cd	0.5 b
Type2/*Wx^b^*	13	13.8 bc	274.0 a	152.1 abc	121.9 a	222.4 bc	−51.6 c	3.6 c	2.1 cd	0.5 b
Type3/*Wx^b^*	13	13.2 c	277.9 a	157.3 abc	120.6 a	241.5 bc	−36.4 c	3.4 c	1.6 d	0.5 b
Type4/*Wx^b^*	6	12.9 c	271.0 a	155.3 abc	115.7 a	235.3 bc	−35.7 c	4.0 c	2.3 cd	0.5 b
Type1/*Wx^lv^*	13	27.3 a	185.6 cd	140.1 c	45.5 c	252.1 bc	66.5 ab	13.1 b	2.9 ab	0.5 b
Type2/*Wx^lv^*	8	25.3 a	191.3 c	140.1 c	51.2 c	264.8 b	73.4 a	14.3 ab	6.2 ab	0.4 b
Type3/*Wx^lv^*	5	26.1 a	198.9 bc	144.4 bc	54.5 bc	247.4 bc	48.5 ab	18.5 a	8.8 a	0.5 b
Type3/*Wx^la/mw^*	5	10.8 c	255.9 a	137.9 c	118.0 a	196.5 bc	−59.4 c	2.9 c	1.4 d	0.5 b
Type2/*wx*	6	1.8 d	202.7 bc	119.6 c	83.1 b	158.1 c	−44.6 c	3.1 c	2.8 cd	0.8 a
Type3/*wx*	19	1.4 d	143.5 d	76.0 d	67.5 bc	96.5 d	−47.0 c	3.0 c	2.6 cd	0.8 a
Type4/*wx*	13	1.4 d	145.1 d	77.6 d	67.6 bc	97.8 d	−47.3 c	4.1 c	3.9 bcd	0.8 a

Different letters in the same column indicated significant difference at *p* < 0.05.

**Table 8:** Thermal properties of rice with different *SSIIa* and *Wx* allele combinations.

Alleles	Number	To (°C)	Tp (°C)	Tc (°C)	ΔHg (J/g)
Type1/*Wx^a^*	21	73.0 a	76.8 a	80.6 ab	5.7 b
Type1/*Wx^lv^*	13	73.4 a	77.4 a	81.6 ab	6.4 b
Type2/*Wx^a^*	14	71.8 ab	76.5 a	81.0 ab	6.5 b
Type2/*Wx^in^*	7	71.5 ab	76.8 a	81.8 ab	6.3 b
Type2/*Wx^b^*	13	73.0 a	77.4 a	82.5 a	6.7 b
Type2/*Wx^lv^*	8	71.5 ab	76.0 ab	81.0 ab	7.2 ab
Type2/*wx*	6	71.1 ab	77.0 a	83.8 a	8.2 a
Type3/*Wx^b^*	13	65.3 e	71.4 d	76.7 d	6.0 b
Type3/*Wx^lv^*	5	69.3 bc	73.9 bc	78.4 cd	6.4 b
Type3/*Wx^la/mw^*	5	68.4 bc	73.9 bc	79.6 bc	6.5 b
Type3/*wx*	19	65.5 e	72.1 cd	77.8 cd	7.0 b
Type4/*Wx^b^*	6	67.4 cde	72.7 cd	77.6 cd	6.0 b
Type4/*wx*	13	67.4 cde	72.3 cd	77.5 cd	6.4 b

Different letters in the same column indicated significant difference at *p* < 0.05.

**Table 9:** Primers required for sequencing of *Wx* and *SSIIa* genes.

Gene	Primer Name	Forward Primer	Reverse Primer	PCR Product Length (bp)
*Wx*	*Wx*1	CCCGTCCCGTTGCGTCGTCAT	CCCGGAGATGCACAGAGCTTTGAG	1113
	*Wx*2	TTAGTAGCCGAGTTGGTCAAAGG	CCACCTTCTCCAGGAATGACG	1356
	*Wx*3	ACCAGTACAAGGACGCTTGG	ATGAGCTCCTCGGCGTAGTA	1175
	*Wx*4	GAGGGCAGGAAGATCAACTG	TCCCCTGCAGCTGGATGAGT	903
	*Wx*5	GAGAAGCTGCTCAAGAGCA	ATTGGCTATAGCTACAAATT	1146
*SSIIa*	*SSIIa*1	GTTTTATACCCCCACCCCC	CGTGCGGGGGATGAAAT	800
	*SSIIa*2	CGGTGGCGCACGCTTTA	CTCTCCTCGCCAAAGCTTTG	1450
	*SSIIa*3	CCAGGATGACGATTCGGG	TGCAGAAACATTTACGGGCTG	1550
	*SSIIa*4	TTTGTAAGGCTGCTGTTGAGG	CTTCCAGTCGTTCTCCCGTAT	1300
	*SSIIa*5	CTACATGGAATTGCCGGAGC	GACCGCATGCTCACAGTTG	1250

## Data Availability

The original contributions presented in this study are included in the article/Supplementary Material. Further inquiries can be directed to the corresponding author.

## Supplementary Materials

The following supporting information can be downloaded at https://www.mdpi.com/article/10.3390/ijms27062588/s1.

## Abbreviations

The following abbreviations are used in this manuscript:

AAC	Apparent amylose content
ADH	Adhesiveness
BD	Breakdown
CEQ	Cooking and eating quality
COH	Cohesiveness
CPV	Cold paste viscosity
DSC	Different scanning calorimetry
GBSS	Granule bound starch synthase
GC	Gel consistency
GUM	Gumminess
HD	Hardness
HPV	Hot paste viscosity
PT	Pasting temperature
PV	Peak viscosity
RVA	Rapid visco analyzer
RVU	Rapid visco unit
SB	Setback
SNP	Single nucleotide polymorphism
Tc	Conclusion temperature
To	Onset temperature
Tp	Peak temperature
ΔHg	Enthalpy of gelatinization
